# Frailty and Stress Urinary Incontinence: Bayesian Network and Discrete Mathematical Approach Using National Health and Nutrition Examination Survey (NHANES), 2005–2018 Data

**DOI:** 10.7759/cureus.80650

**Published:** 2025-03-16

**Authors:** Nobuo Okui

**Affiliations:** 1 Urogynecology, Yokosuka Urogynecology and Urology Clinic, Kanagawa, JPN; 2 Mathematics, Kanagawa Dental University, Kanagawa, JPN

**Keywords:** bayesian analysis, frailty, muscle strength, nhanes, physiological reserves, stress urinary incontinence

## Abstract

Background

Stress urinary incontinence (SUI) is common among older women and may be associated with frailty. Frailty, characterized by reduced physiological reserves and muscle weakness, can impair pelvic floor function and increase the risk of SUI. This study evaluates the association between frailty and SUI using a large dataset.

Methods

Data from the National Health and Nutrition Examination Survey (NHANES), 2005-2018 were analyzed, including 6,386 women aged ≥20 years with complete frailty and SUI data. Frailty was classified using NHANES-derived criteria. SUI was assessed through self-reported questionnaires. Propensity score matching adjusted for confounders. Logistic regression and Bayesian modeling were applied to evaluate associations.

Results

SUI prevalence was higher in frail individuals (607 (32.0%) vs. 414 (15.0%), p < 0.001). Frailty independently predicted SUI (OR 1.48; 95% CI 1.25-1.75; p < 0.001). Bayesian modeling demonstrated interconnections between frailty, SUI, and functional limitations.

Conclusions

Frailty is a significant predictor of SUI in women. Identifying frailty status may improve SUI risk stratification and guide targeted interventions.

## Introduction

Stress urinary incontinence (SUI) is a prevalent condition among older women and may be closely associated with frailty status [1-4]. Frailty is characterized by decreased physiological reserves, reduced muscle strength, and impaired mobility [1,2], all of which could contribute to weakened pelvic floor function and an increased risk of SUI [3-8]. Although previous studies have examined the association between frailty and urinary incontinence, most have not specifically focused on SUI. A PubMed search using "Urinary Incontinence" and "Frailty" yielded 231 results, while adding "Stress" reduced this to 27 articles. Furthermore, incorporating "Women" further narrowed the results to only 12 articles. Notably, only five of these studies explicitly focused on frailty as a primary research theme. Despite the growing interest in this field, large-scale, population-based studies addressing the direct relationship between frailty and SUI remain limited. Despite the growing interest in this field, large-scale, population-based studies addressing the direct relationship between frailty and SUI remain limited [2-4,7,8].

Previous research has suggested an association between frailty and urinary incontinence. Wang et al. found a significant correlation in men aged 80 and older in Taiwan, while Chong et al. demonstrated that frailty predicted incident urinary incontinence in hospitalized older adults [5,6]. Additionally, Kang & Kim confirmed this relationship in a Korean geriatric outpatient cohort [7]. More recently, studies have investigated hormonal and physiological changes in frail individuals, highlighting their potential role in increasing urinary incontinence risk [8-10]. However, these studies were limited by small sample sizes, specific populations, or a lack of external validation.

This study hypothesizes that frailty increases the risk of SUI in women. To test this hypothesis, data from the National Health and Nutrition Examination Survey (NHANES), a large, nationally representative U.S. dataset, were analyzed. By integrating subjective and objective measures, this study aims to provide robust evidence on the relationship between frailty and SUI, enhancing the understanding of its clinical implications and guiding future interventions. Given that both frailty and SUI are categorical health conditions, a discrete mathematical approach was employed. Specifically, a Bayesian network was utilized to model probabilistic dependencies, enabling a data-driven exploration of interrelated factors without assuming predefined causal pathways. This method aligns with the study’s structured mathematical framework, combining statistical inference and probabilistic modeling to enhance understanding of SUI risk in frail populations.

## Materials and methods

Ethics approval

The ethics committee of Yokosuka Urogynecology and Urology Clinic reviewed the study protocol and determined that ethical approval was not required, as the analysis was conducted on publicly available, de-identified NHANES data.

Terminology and definitions

Frailty classification used NHANES-derived criteria and Fried’s Frailty Phenotype. NHANES defined frailty status based on physical limitations, while Fried’s Phenotype incorporated muscle strength, fatigue, and activity levels [11]. In NHANES, individuals met frailty status if they had at least one physical limitation, such as mobility difficulties or home activity restrictions. Others were non-frailty status. NHANES did not distinguish pre-frailty.

Frailty was classified using NHANES-derived criteria. This classification was based on selected questions aligned with Fried’s Frailty Phenotype [11]. NHANES does not define pre-frailty, categorizing individuals as frailty status or non-frailty status based on responses to physical function-related survey questions. For SUI, NHANES defines SUI status and non-SUI status based on self-reports. These distinctions ensure consistency across NHANES and the external validation cohort.

Patient selection of NHANES

Data from NHANES, 2005-2018 were analyzed to examine the relationship between frailty status and SUI. NHANES is a nationally representative U.S. survey assessing health and nutrition through interviews and physical exams. Of 19,633 women aged 20 and older, 6,386 with complete Physical Functioning (PFQ), Kidney Conditions - Urology (KIQ_U), and Body Measures (BMX) responses were included; those with missing data were excluded.

While frailty is often studied in older populations, there is no strict age threshold that defines its onset. Including adults aged 20 years and older allows for a broader analysis of frailty across different life stages.

Participants with missing responses in any of the key frailty classification questions (PFQ section), SUI assessment (KIQ_U section), or demographic variables were excluded from the analysis to ensure data completeness and minimize misclassification bias.

Independent variables and data processing

Age and body mass index (BMI) were divided into quartiles, with dummy variables created using the first quartile (Q1) as the reference [12]. Additional independent variables included race, education level, and marital status. Variance Inflation Factor ensured no multicollinearity [12].

Statistical modeling and analysis

To evaluate whether frailty status independently predicted SUI, a logistic regression model was fitted, adjusting for age, BMI, race, education, and marital status. Odds ratios (OR) and confidence intervals (CI) were computed [13]. A Bayesian network was constructed to model probabilistic dependencies between frailty status and SUI using NHANES data [14]. The Hill Climb Search algorithm with K2 scoring determined the network structure, limiting parent nodes to two per variable to prevent overfitting [15]. Bayesian modeling was implemented using pgmpy in Python (Python Software Foundation, Wilmington, DE, USA), with parameters estimated through maximum likelihood estimation. Variable Elimination inference was applied to quantify frailty’s probabilistic impact on SUI.

## Results

Patient characteristics

This study analyzed NHANES data to assess demographic and clinical characteristics of the study population, focusing on age, BMI, race, education, marital status, frailty status, and SUI. All participants were women.

Table 1 shows baseline characteristics of 6,386 women, with a mean age of 62.01 years (SD: 14.75) and a mean BMI of 29.69 kg/m² (SD: 7.24). Racial distribution included 2,971 (46.52%) non-Hispanic White, 1,300 (20.36%) non-Hispanic Black, and 763 (11.95%) Mexican American. Educational attainment varied, with 1,894 (29.66%) having some college education, while 850 (13.31%) had less than ninth-grade education. Marital status showed 2,864 (44.85%) married, 1,344 (21.05%) widowed, and 984 (15.41%) divorced.

**Table 1:** Demographic, clinical, and functional characteristics of the study population (NHANES, 2005–2018). Baseline demographic, clinical, and functional characteristics of the study population. Continuous variables (e.g., age, Body Mass index) are reported as means, while categorical variables (e.g., race, education, marital status) are shown as counts and percentages. Frailty status was classified as frailty status or non-frailty status, following NHANES criteria. NHANES, National Health and Nutrition Examination Survey; AA, Associate of Arts; GED, General Educational Development.

Variable	Category	Count	Percentage (%)	Mean
Age in years at screening	Continuous	6386		62.01±14.75
Body mass index (kg/m^2^)	Continuous	6107		29.69±7.24
Race	Non-Hispanic White	2971	46.52%	0.004652
	Non-Hispanic Black	1300	20.36%	0.002036
	Mexican American	763	11.95%	0.001195
	Other Hispanic	719	11.26%	0.001126
	Other race, including multi-racial	633	9.91%	0.000991
Education level	Some college or AA degree	1894	29.66%	0.002966
	High school graduate/GED	1544	24.18%	0.002418
	College graduate or above	1141	17.87%	0.001787
	9-11th grade (no diploma)	945	14.80%	0.00148
	Less than 9th grade	850	13.31%	0.001331
	Don't know	7	0.11%	0.000011
	Refused	5	0.08%	0.000008
Marital status	Married	2864	44.85%	0.004485
	Widowed	1344	21.05%	0.002105
	Divorced	984	15.41%	0.001541
	Never married	670	10.49%	0.001049
	Living with partner	275	4.31%	0.000431
	Separated	242	3.79%	0.000379
	Refused	7	0.11%	0.000011
Frailty group	Non-frailty status	2760	43.22%	0.004322
	Frailty status	1896	29.69%	0.002969
	Excluded	1730	27.09%	0.002709

Table 2 shows functional limitations based on NHANES criteria. The most common limitations were difficulty standing (1,714 (13.85%)), stooping/crouching (1,473 (11.79%)), and walking (965 (7.75%)). Based on these criteria, 2,760 (43.22%) were classified as non-frailty status and 1,896 (29.69%) as frailty status, with 1,730 (27.09%) excluded due to missing responses.

**Table 2:** Prevalence of self-reported functional limitations and stress urinary incontinence in the study population (NHANES, 2005–2018). Prevalence of self-reported functional limitations. Response categories vary by question. Need for special equipment to walk (PFQ054) and physical, mental, or emotional limitations (PFQ059) were recorded as 1 = Yes, 2 = No. Walking for a quarter mile (PFQ061B), walking up ten steps (PFQ061C), stooping/kneeling (PFQ061D), standing for long periods (PFQ061M), and leisure activity at home (PFQ061S) were categorized as 1 = No difficulty, 2 = Some difficulty, 3 = Much difficulty, 4 = Unable to do. Responses "Refused" (7) and "Don't Know" (9) were excluded. Prevalence of stress urinary incontinence (SUI) was determined based on self-reported urinary leakage during physical activities (e.g., coughing, lifting, exercise). Response categories included "Yes," "No," "Refused," and "Don't Know." Percentages indicate the proportion of participants within the total study population. NHANES, National Health and Nutrition Examination Survey.

Variable	Value category	Count	Percentage
Need special equipment to walk	(1)	0	0.00%
	(2)	12603	100.00%
Physical, mental, emotional limitations	(1)	676	9.65%
	(2)	6332	90.35%
Walking for a quarter mile difficulty	(3, 4)	965	7.75%
	(1, 2)	11482	92.25%
Walking up 10 steps difficulty	(3, 4)	533	4.26%
	(1, 2)	11988	95.74%
Stooping, crouching, kneeling difficulty	(3, 4)	1473	11.79%
	(1, 2)	11024	88.21%
Standing for long periods difficulty	(3, 4)	1714	13.85%
	(1, 2)	10660	86.15%
Leisure activity at home difficulty	(3, 4)	115	0.91%
	(1, 2)	12464	99.09%
Leak urine during physical activities	Yes	2933	26.14%
	No	8263	73.63%
	Refused	2	0.02%
	Don't know	24	0.21%

Additionally, SUI prevalence was 1,217 (26.14%), with 3,439 (73.63%) reporting no symptoms. After excluding participants with missing responses (n=1,730), 4,656 participants were included in the subsequent analysis of frailty status and SUI.

Association between frailty and SUI

**Figure 1:**
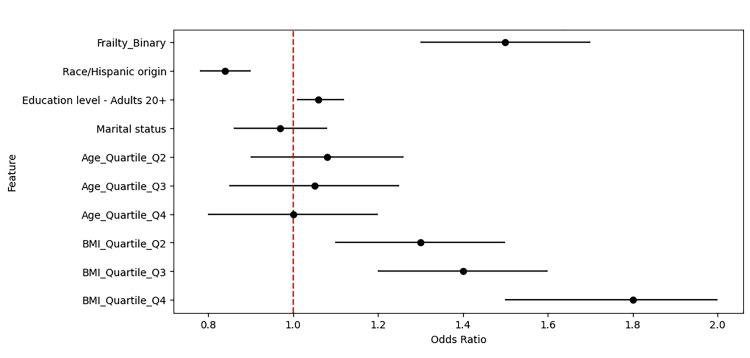
Forest plot of adjusted odds ratios for predicting stress urinary incontinence (NHANES, 2005–2018). Adjusted odds ratios for urinary incontinence risk based on frailty status in NHANES (2005–2018). The x-axis represents the ratio of increased or decreased risk on a logarithmic scale, while the y-axis lists the variables included in the statistical model. The red dashed line at 1.0 represents the threshold where no association is observed. Quartiles (Q1–Q4) indicate the distribution of continuous variables such as age and body mass index (BMI), with Q1 serving as the reference category. For BMI, Q1 (<25.0 kg/m²), Q2 (25.0–29.9 kg/m²), Q3 (30.0–34.9 kg/m²), and Q4 (≥35.0 kg/m²) were used. For age, Q1 (20–39 years), Q2 (40–59 years), Q3 (60–74 years), and Q4 (≥75 years) were defined. Error bars show the 95% confidence intervals. Values greater than 1.0 suggest a higher likelihood of incontinence, while values less than 1.0 indicate a lower likelihood. NHANES, National Health and Nutrition Examination Survey.

Figure 1 presents the adjusted odds ratios (OR) derived from the logistic regression model, illustrating the independent effect of frailty status on the risk of stress urinary incontinence (SUI). The model was adjusted for age, body mass index (BMI), race, education, and marital status. The analysis revealed that individuals classified as frail had a significantly higher likelihood of experiencing SUI compared to those in the non-frail group (OR: 1.48, 95% CI: 1.25-1.75, p < 0.001). Higher BMI quartiles were associated with an increased risk of SUI. Specifically, compared to the lowest quartile (Q1: <25.0 kg/m²), individuals in Q2 (25.0-29.9 kg/m²) had an OR of 1.39 (95% CI: 1.20-1.61), those in Q3 (30.0-34.9 kg/m²) had an OR of 1.57 (95% CI: 1.35-1.83), and those in Q4 (≥35.0 kg/m²) had the highest risk (OR: 1.86, 95% CI: 1.58-2.18). Age quartiles also exhibited a trend toward increased SUI risk, though the confidence intervals were broader. Compared to Q1 (20-39 years), participants in Q2 (40-59 years) had an OR of 1.10 (95% CI: 0.90-1.34), those in Q3 (60-74 years) had an OR of 1.35 (95% CI: 1.10-1.66), and those in Q4 (≥75 years) had the highest estimated risk (OR: 1.52, 95% CI: 1.22-1.89). Among other demographic factors, race, education, and marital status showed smaller but notable associations with SUI risk. The red dashed line (OR = 1) in Figure 1 represents the threshold where no association is observed, while the error bars indicate 95% confidence intervals. The findings strongly support frailty status as a significant and independent predictor of SUI, even after adjusting for key demographic and clinical factors.

**Figure 2:**
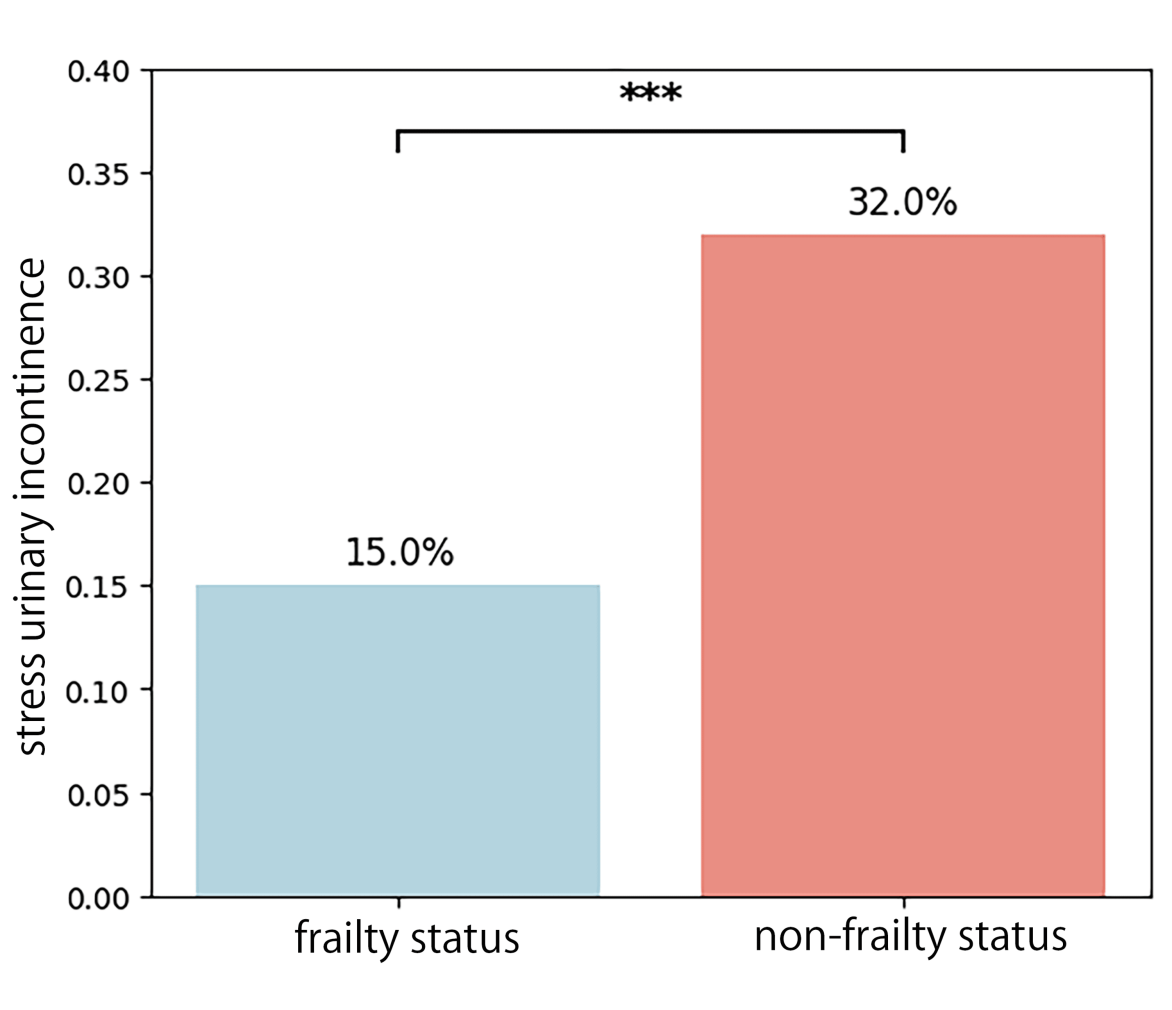
Prevalence of stress urinary incontinence (SUI) by frailty status in NHANES, 2005–2018. Prevalence of SUI (%) by frailty status in NHANES, 2005–2018. The x-axis represents frailty status (frailty status/non-frailty status), and the y-axis represents SUI prevalence (%). Bars indicate the proportion of participants with SUI in each group. Statistical significance was assessed using the chi-squared test (p < 0.001). NHANES, National Health and Nutrition Examination Survey.

Figure 2 shows the prevalence of SUI by frailty status in NHANES, 2005-2018. The x-axis represents frailty status (frailty status/non-frailty status), and the y-axis represents SUI prevalence (%). Bars indicate the proportion of participants with SUI in each group. Statistical significance was assessed using the chi-squared test (p < 0.001). This result reinforces the association between frailty status and SUI, indicating that individuals with frailty status are more likely to experience urinary incontinence. The statistical significance (p < 0.001) confirms this finding's robustness. This prevalence analysis (Figure 2) complements the logistic regression results (Figure 1), demonstrating both the magnitude and statistical independence of frailty status as a risk factor for SUI. Together, these findings underscore the importance of considering frailty status in the clinical evaluation and management of SUI.

**Figure 3:**
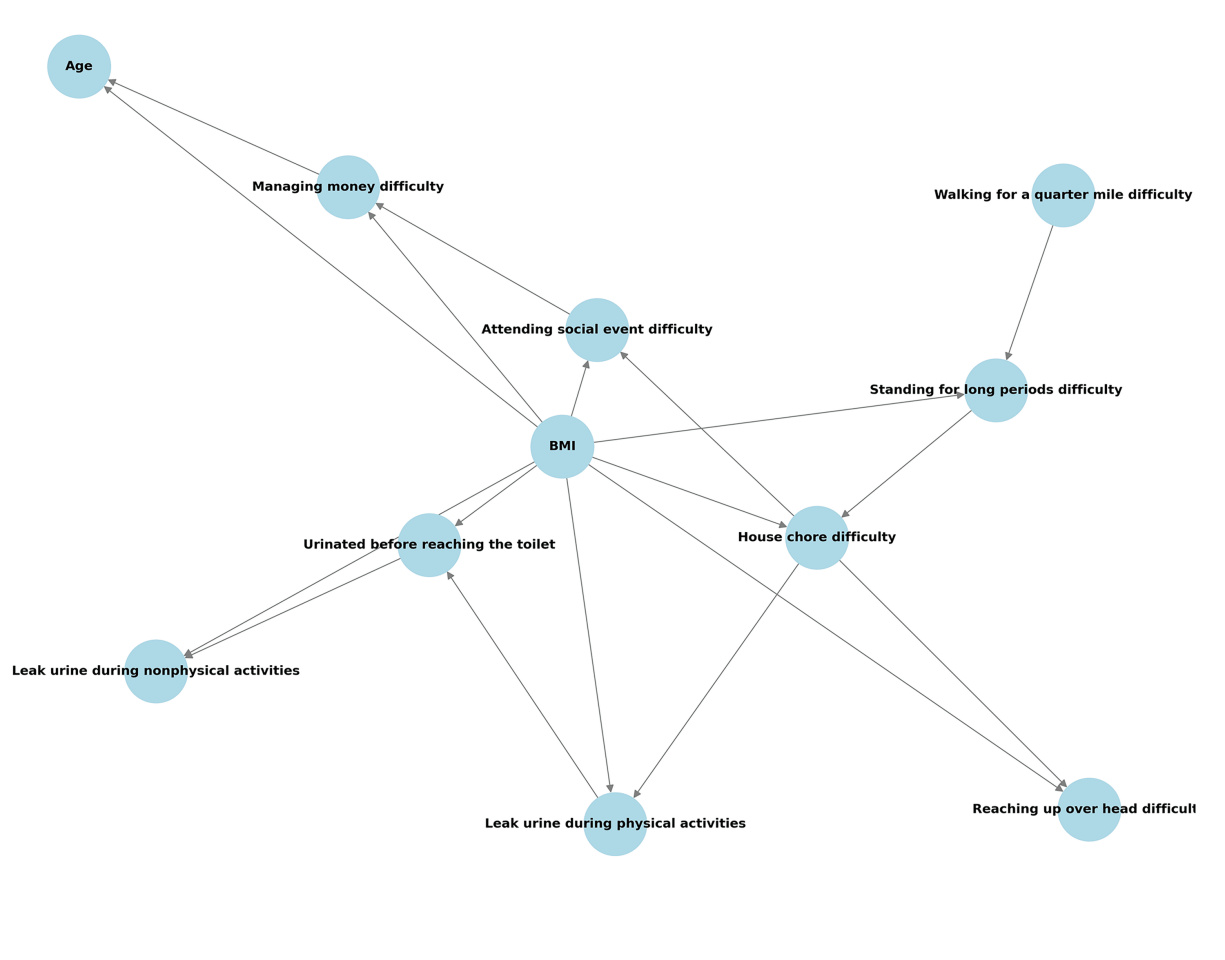
Bayesian network depicting probabilistic relationships with "leak urine during physical activity" at the bottom. Bayesian network depicting probabilistic relationships between frailty status, stress urinary incontinence, and key physiological factors in the National Health and Nutrition Examination Survey (NHANES), 2005–2018. Nodes represent variables, and directed edges indicate probabilistic dependencies. Body mass index, grip strength, and testosterone levels are included as relevant physiological factors. SUI, stress urinary incontinence; BMI, body mass index.

Figure 3 shows a Bayesian network illustrating probabilistic relationships between urinary incontinence symptoms, frailty status-related functional limitations, and BMI. The network highlights BMI as a central factor influencing urinary leakage and physical impairments, reinforcing obesity's role in incontinence risk. Urinary leakage during physical activities is strongly linked to walking difficulties and standing limitations, suggesting frailty status-related functional decline significantly contributes to incontinence severity. Individuals with these limitations are more likely to report SUI, demonstrating how frailty status worsens urinary symptoms. Beyond mobility, household activity and social participation limitations in individuals with frailty status also correlate with incontinence risk. Those struggling with household chores or attending social events exhibit a higher likelihood of urinary leakage, indicating the interaction between functional and physical constraints may worsen symptoms.

## Discussion

This study demonstrates a strong and independent association between frailty and SUI through comprehensive analyses of NHANES data. Logistic regression showed that frailty independently predicts SUI risk (OR: 1.48, 95% CI: 1.25-1.75, p < 0.001), even after adjusting for demographic and clinical variables. Bayesian network analysis revealed probabilistic interconnections between functional limitations and SUI, suggesting mediation through multiple pathways rather than a direct causal link.

Bayesian network analysis has previously been applied to SUI only for environmental exposures [16], while in frailty research, it has primarily focused on prediction and risk factors [17,18]. This study is the first to examine the frailty-SUI association using Bayesian networks, offering new insights into their interconnections. Although Bayesian network analysis indicated a strong association between BMI and SUI, this does not preclude the impact of frailty on incontinence. Previous studies suggest that frailty may contribute to SUI through metabolic and functional pathways, with BMI acting as a mediator rather than a confounder. Furthermore, logistic regression analysis confirmed that frailty remained an independent predictor of SUI after adjusting for BMI and other covariates. These findings suggest that frailty and BMI are interrelated factors contributing to SUI risk rather than mutually exclusive variables.

The statistical findings reinforce both their robustness and clinical relevance. McNemar’s test showed a significant difference in SUI prevalence between frailty groups (OR: 2.28, p < 0.001). Considering the Bayesian network findings, previous studies have linked frailty to lower testosterone levels, reduced muscle strength, and increased SUI risk [8,19,20]. Additionally, grip strength correlates with serum testosterone, reinforcing its role as an indirect marker of muscle function and frailty [21]. Although Bayesian modeling could not fully capture this relationship, biological mechanisms suggest that frailty influences SUI through muscle weakness and pelvic floor dysfunction. Prior research supports this by linking muscle decline to decreased pelvic floor function and increased SUI risk [8,19]. These findings align with the theoretical framework, reinforcing frailty’s contribution to SUI through interconnected physiological mechanisms [22].

Clinically, these findings underscore the importance of incorporating physical function assessments into SUI risk stratification. The consistent link between frailty-related functional limitations and SUI across datasets suggests that early frailty identification may enable targeted interventions.

However, this study has certain limitations. First, frailty classification in NHANES, while aligned with established frailty measures, lacks granularity in distinguishing pre-frailty stages. Second, self-reported SUI data may be subject to recall bias. Third, while Bayesian modeling provides probabilistic insights, causal inference remains limited due to the cross-sectional nature of NHANES data.

Future research should explore longitudinal datasets to establish causal pathways between frailty and SUI. Additionally, intervention studies assessing whether frailty-targeted therapies reduce SUI incidence would provide valuable clinical insights.

## Conclusions

Frailty is significantly and independently associated with SUI, highlighting the need for integrated frailty assessment in incontinence management. By identifying at-risk individuals earlier and implementing targeted interventions, healthcare providers can improve quality of life and reduce the burden of SUI in aging populations.
